# An *Axin2* mutation and perinatal risk factors contribute to sagittal craniosynostosis: evidence from a Chinese female monochorionic diamniotic twin family

**DOI:** 10.1186/s41065-021-00182-0

**Published:** 2021-06-16

**Authors:** Jin Xu, Qing Yan, Chengcheng Song, Jingjia Liang, Liang Zhao, Xin Zhang, Zhenkun Weng, Cheng Xu, Qian Liu, Shuqin Xu, Lu Pang, Liye Zhang, Yuan Sun, Gang Wang, Aihua Gu

**Affiliations:** 1 State Key Laboratory of Reproductive Medicine, Institute of Toxicology, Nanjing Medical University, Nanjing, 211166 China; 2 Key Laboratory of Modern Toxicology, Ministry of Education, Center for Global Health, School of Public Health, Nanjing Medical University, Nanjing, 211166 China; 3 Department of Maternal, Child and Adolescent Health, School of Public Health, Nanjing Medical University, Nanjing, 211166 China; 4 Department of Neurosurgery, Children’s Hospital of Nanjing Medical University, Nanjing, 210017 China; 5 Obstetrics and Gynecology Hospital, NHC Key Laboratory of Reproduction Regulation (Shanghai Institute of Planned Parenthood Research), School of Life Sciences, Shanghai, 200011 China; 6 Department of Neurosurgery, The First Affiliated Hospital of Nanjing Medical University, Nanjing, 210029 China

**Keywords:** Sagittal craniosynostosis, *Axin2*, Perinatal risk factors, Mutation, Monochorionic diamniotic twin

## Abstract

**Background:**

Craniosynostosis, defined as premature fusion of one or more cranial sutures, affects approximately 1 in every 2000–2500 live births. Sagittal craniosynostosis (CS), the most prevalent form of isolated craniosynostosis, is caused by interplay between genetic and perinatal environmental insults. However, the underlying details remain largely unknown.

**Methods:**

The proband (a female monochorionic twin diagnosed with CS), her healthy co-twin sister and parents were enrolled. Obstetric history was extracted from medical records. Genetic screening was performed by whole exome sequencing (WES) and confirmed by Sanger sequencing. Functional annotation, conservation and structural analysis were predicted in public database. Phenotype data of *Axin2* knockout mice was downloaded from The International Mouse Phenotyping Consortium (IMPC, http://www.mousephenotype.org).

**Results:**

Obstetric medical records showed that, except for the shared perinatal risk factors by the twins, the proband suffered additional persistent breech presentation and intrauterine growth restriction. We identified a heterozygous mutation of *Axin2* (c.1181G > A, p.R394H, rs200899695) in monochorionic twins and their father, but not in the mother. This mutation is not reported in Asian population and results in replacement of Arg at residue 394 by His (p.R394H). Arg 394 is located at the GSK3β binding domain of *Axin2* protein, which is highly conserved across species. The mutation was predicted to be potentially deleterious by in silico analysis. Incomplete penetrance of *Axin2* haploinsufficiency was found in female mice.

**Conclusions:**

*Axin2* (c.1181G > A, p.R394H, rs200899695) mutation confers susceptibility and perinatal risk factors trigger the occurrence of sagittal craniosynostosis. Our findings provide a new evidence for the gene-environment interplay in understanding pathogenesis of craniosynostosis in Chinese population.

**Supplementary Information:**

The online version contains supplementary material available at 10.1186/s41065-021-00182-0.

## Introduction

Craniosynostosis (CS), defined as premature fusion of one or more cranial sutures, affects approximately 1 in every 2000–2500 live births [1]. CS contains an isolated condition (non-syndromic craniosynostosis, NCS) and complex syndromes (with other malformations, syndromic craniosynostosis) [2]. Sagittal craniosynostosis is the most prevalent form of NCS, accounting for 40–58% of all NCS cases [3]. Sagittal suture premature closure restricts the widen of the skull and then causes the scaphocephaly deformity and other adverse neurologic outcomes [4].

Pathoetiology of NCS involves interplay between genetic and environmental factors [5–8]. FGF (fibroblast growth factor), BMP (bone morphogenic protein), Wnt (wingless-type integration sites) pathways are major regulators in suture biology [9, 10]. Pivotal component mutations in these pathways, including *FGFR2* (fibroblast growth factor receptor 2), *TWIST1* (twist, drosophila, homolog of 1) and *Axin2* (axis inhibitor 2), have been regarded as the origin of craniosynostosis [1, 9]. Non-genetic risk factors, like intrauterine constraint, twin gestation, breech delivery, low birth weight, malnutrition, premature delivery, maternal thyroid disorders, gestational diabetes, virus infectious, can either cause or exacerbate craniosynostosis [7, 11–14]. Although several findings demonstrate the interactions between genetic and environmental risk factors contribute to premature fusion of cranial sutures [5, 15], more evidence are still need.

Monochorionic (MC) twins, sharing almost the same genome, offer a unique opportunity to study the gene-environment interactions, for the healthy twin as an ideal control. Discordant phenotypes between MC twins emphasize the interplay between genetic and environmental influences in etiologies of the disease [16].

In our study, we found that a heterozygous mutation of *Axin2* (c.1181G > A, p.R394H, rs200899695) exist in Chinese female monochorionic sisters and their father. However, only the proband, suffering a persistent breech presentation and intrauterine growth restriction, was diagnosed with sagittal craniosynostosis. Public database indicated that *Axin2* (c.1181G > A, p.R394H, rs200899695) mutation was not detected in Asian population. According to data from The International Mouse Phenotyping Consortium (IMPC, http://www.mousephenotype.org), about 22% female *Axin2* heterozygous knockout mice developed abnormal head shape before delivery. Thus, we speculate that this particular *Axin2* mutation leads to haploinsufficiency in female with incomplete penetrance, and additional environmental insults eventually trigger the occurrence of sagittal craniosynostosis.

## Materials and methods

### Clinic Examination and Information

All participants signed the informed consent and received physical examination by two experienced surgeons in Children’s Hospital of Nanjing Medical University. All samples used in our study were in compliance with the informed consent and agreement of patients. This study was approved by the ethics committee of Nanjing Medical University.

Clinical information of pregnancy history and infant clinical data were obtained from hospital medical records. Head CT scan of the healthy child was performed upon the request of the parents.

### Whole Exome Sequencing and Data Analysis

**Fig. 1:**
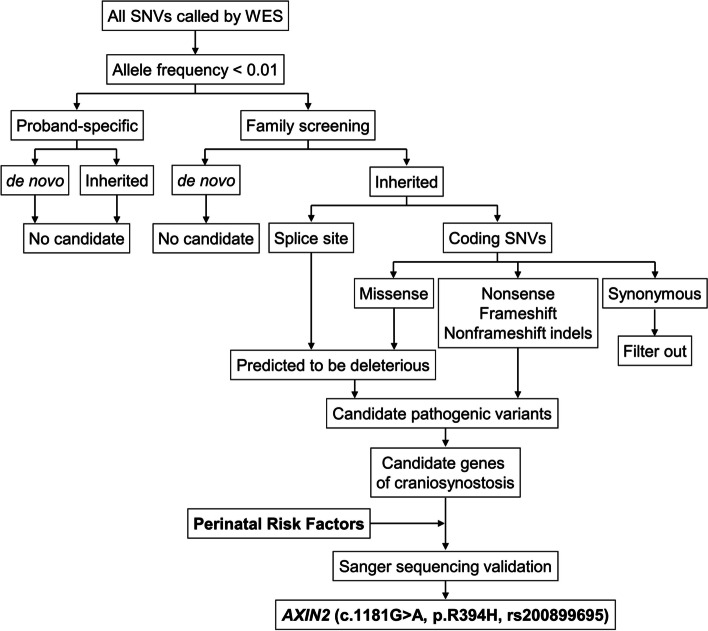
Workflow for identifying *Axin2* (c.1181G > A, p.R394H, rs200899695) mutation in pedigree diagnosed with sagittal craniosynostosis

Genomic DNA, extracted from peripheral blood samples (proband, proband’s co-twin sister, the parents) and proband’s skull periosteum tissue, was subjected to whole-exome sequencing (WES) on the platform of Genergy Biotechnology, Shanghai, China. Raw reads were aligned to the human genome reference assembly (GRCh37/hg19) using the Burrows-Wheeler Aligner [17]. The Picard software was employed to remove PCR duplicates and evaluate the quality of variants. DNA variants was called and analyzed using the Genome Analysis Toolkit [18]. The variants with read depths less than 4 × were filtered out. All variants were further annotated [19–25]. The workflow of genetic analysis was shown in Fig. 1.

### Sanger Sequencing

*Axin2* mutation was validated by Sanger sequencing in Tsingke (China) using the following primers: Forward: 5′-CGCACACCCTAACGCACCCCAT-3′ and Reverse: 5′-ACCGCCCACCTAGCCTGCTGAA-3′. Results were visualized using FinchTV (Geospiza) software.

### Conservation Analysis

Multiple-species amino acid sequences were obtained from National Center for Biotechnology Information (NCBI), and were analyzed by WebLogo (Version 2.8.2, http://weblogo.berkeley.edu)[26].

### Structural Analysis

Three-dimensional models of the wild-type and mutant *Axin2* protein were constructed by I-TASSER [27] and visualized using the PyMOL software (PyMOL Molecular Graphics System, DeLano Scientific, San Carlos, CA).

### Functional Annotation

Functional annotation of *Axin2* (c.1181G > A: p.R394H, rs200899695) was conducted on FAVOR functional annotation online portal (http://www.favor.genohub.org/) [28] or the University of California, Santa Cruz Genome Browser (UCSC, http://www.genome.ucsc.edu).

### Phenotype analysis of *Axin2* knockout mice

Phenotypes of *Axin2* homozygous (*Axin2*^−/−^) and heterozygous (*Axin2*^*+/−*^) knockout mice were obtained from The International Mouse Phenotyping Consortium (IMPC, http://www.mousephenotype.org) [29, 30].

## Results

### Clinical information

**Fig. 2:**
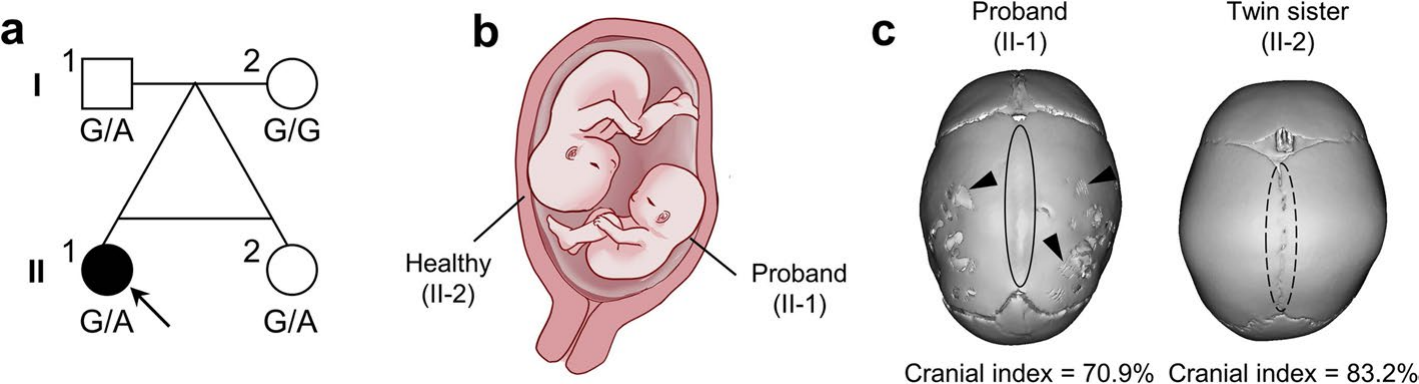
Clinical features of the pedigree with sagittal craniosynostosis. **a** Pedigree of the family. Proband is marked with an upward arrow. Open squares/circles denote unaffected individuals; squares denote males and circles denote females. **b** Schematic diagram of fetal position before delivery. Proband (II-1) was in breech presentation at left side of the uterus, while her healthy sister (II-2) was in cephalic presentation at right side of the uterus. **c** Computerized tomographic (CT) scan revealed premature closure of sagittal suture (solid circle) and digital impressions (arrowheads) in proband’s head (left panel). Cranial index of the proband is 70.9%. The co-twin sister was also received CT examination under the request of the parents. Her sagittal suture remains patent (dotted circle) and cranial index is 83.2% (right panel)

The pedigree came from Wuhan, Hubei Province, China. Female proband (II-1), the elder monochorionic diamniotic (MCDA) twin (Fig. 2a-c and Supplementary Fig. S1-3), was diagnosed sagittal craniosynostosis at the age of 9 months in the Department of Neurosurgery, Children’s Hospital of Nanjing Medical University. She was born to non-consanguineous parents without family history of craniosynostosis. Her mother, a 30-year-old Chinese female, conceived MCDA twins by frozen embryo transfer (FET) (Supplementary Fig. S1-3). Her father was 36-year-old at that time. Twenty-six days after FET, two embryos inside a gestational sac (approximately 22 mm × 13 mm) was confirmed by four-dimensional ultrasound scan (Supplementary Fig. S1). Embryo length were 2.8 mm and 3.3 mm, respectively. Fetal heart rate were 107 per minute and 118 per minute, respectively (Supplementary Fig. S1).

At 13 weeks of gestation, IgG of cytomegalovirus and herpes simplex virus were detected in the mother’s serum (Supplementary Table S1). At 14 weeks of gestations, dietary assessment indicated that the mother had inadequate intakes of energy, protein, fat, several vitamins and minerals, while excessive consumption of carbohydrate (Supplementary Table S2). At 17 weeks of gestations, decreased thyroid-stimulating hormone (TSH) level and increased level of urinary iodine were detected (Supplementary Table S3). At 27 weeks of gestations, the mother was diagnosed with gestational diabetes mellitus (Supplementary Table S3).

The ultrasound scan detection indicated that the proband (II-1) had been in persistent breech position on the left side of uterus, while the younger sister (II-2) had been in cephalic position (Fig. 2a, b). At 28 weeks of gestations, the proband (II-1) and twin younger sister (II-2) were born via spontaneous vaginal delivery (Fig. 2a, b). Birth weight of proband (II-1) and co-twin (II-2) was 880 g (ranking 50^th^ -90^th^ centiles for postnatal weight of infants [31]) and 990 g (ranking 90^th^ -97^th^ centiles for postnatal weight of infants [31]), respectively. The proband (II-1) were diagnosed with sagittal craniosynostosis and intrauterine growth restriction (Fig. 2c), while no signs of craniofacial deformity were detected in parents (I-1, I-2) and co-twin sister (II-2) (Fig. 2c). Cranial index, which represents the ratio of maximum cranial width to maximum cranial length, is decreased in patients with sagittal craniosynostosis [32]. In our case, the cranial index of the proband and co-twin sister was 70.9%, 83.2%, respectively. In addition, tooth agenesis, oral clefts or colorectal cancer were not detected in this family through physical examination by clinical doctor as well as medical history inquiry. And the other family members have no history of tooth agenesis, oral clefts or colorectal cancer. Taken together, clinical records indicate that the proband suffered sagittal craniosynostosis, persistent breech presentation and intrauterine growth restriction, except for other shared perinatal risk factors of the twins.

### Mutation analysis of *AXIN2* (c.1181G > A, p.R394H, rs200899695)

**Fig. 3:**
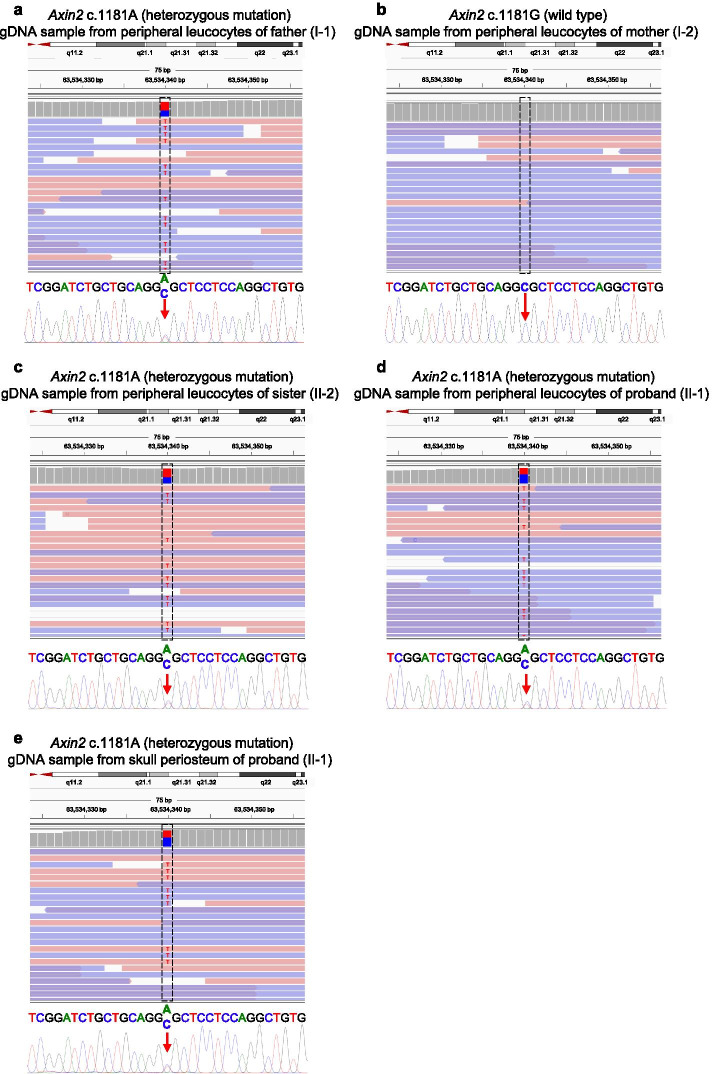
Sequence analysis of *Axin2* (c.1181G > A, p.R394H, rs200899695). **a-e** Integrative Genomics Viewer (IGV) of the sequences around *Axin2* (c.1181G > A, p.R394H, rs200899695) and the results of Sanger sequencing were shown. The gDNA samples were extracted from peripheral leucocytes of the parents and twin sisters (**a**-**d**) and from skull periosteum tissue of the proband (**e**). Please note that the DNA sequences are shown in + strand, but *Axin2* is located on-strand. The father and two twins have this missense mutation (**a**, **c**, **d**, **e**), but the mother does not carry this mutation (**b**)

Whole-exome sequencing was applied to identify the potential genetic etiology leading to sagittal craniosynostosis in our case. Due to the low incidence rate of sagittal craniosynostosis [3], we focused on private and/or rare (minor allele frequency, MAF < 0.01) variants on exons or splicing sites (Fig. 1). However, none of candidate germline or somatic mutations were proband-specific. As gene-environment interactions have been demonstrated in the pathogenesis of craniosynostosis [5, 15], we wonder whether the intrauterine risk exposures (environment factors) triggered the susceptible individual to develop sagittal craniosynostosis. Based on this hypothesis, we re-analyzed our sequencing data and identified a heterozygous missense mutation of *Axin2* (c.1181G > A, p.R394H, rs200899695) in the leukocytes of subjects I-1, II-1 and II-2, and skull periosteum tissue of subject II-1. This finding was further validated by Sanger sequencing (Fig. 3a-e). The frequency of *Axin2* (c.1181G > A, p.R394H, rs200899695) mutation of global population is 0.000849, 0.000637, 0.00008 in gnomAD, ExAC, ALFA database, respectively, while is not detected in Asian population (Table 1).

**Table 1:** Allele frequency of *Axin2* (c.1181G > A: p.R394H, rs200899695) in human populations (dbGaP, Release Version: 20,200,227,123,210)

Study	Population	Sample size	Ref Allele	Alt Allele	BioProject ID	BioSample ID
**gnomAD—Exomes**	Global	250,890	G = 0.999151	A = 0.000849	PRJNA398795	SAMN07488253
gnomAD—Exomes	European	134,850	G = 0.999993	A = 0.000007		SAMN10181265
gnomAD—Exomes	Asian	49,008	G = 1.00000	A = 0.00000		
gnomAD—Exomes	American	34,584	G = 0.99396	A = 0.00604		SAMN07488255
gnomAD—Exomes	African	16,244	G = 1.00000	A = 0.00000		SAMN07488254
gnomAD—Exomes	Ashkenazi Jewish	10,072	G = 1.00000	A = 0.00000		SAMN07488252
gnomAD—Exomes	Other	6132	G = 0.9995	A = 0.0005		SAMN07488248
**ExAC**	Global	120,894	G = 0.999363	A = 0.000637	PRJEB8661	SAMN07490465
ExAC	Europe	72,950	G = 0.99999	A = 0.00001		
ExAC	Asian	25,152	G = 1.00000	A = 0.00000		
ExAC	American	11,542	G = 0.99342	A = 0.00658		
ExAC	African	10,358	G = 1.00000	A = 0.00000		
ExAC	Other	892	G = 1.000	A = 0.000		SAMN07486028
**ALFA**	Total	62,874	G = 0.99992	A = 0.00008	PRJNA507278	SAMN10492705
ALFA	European	59,864	G = 0.99995	A = 0.00005		SAMN10492695
ALFA	Other	2654	G = 0.9992	A = 0.0008		SAMN11605645
ALFA	African	242	G = 1.000	A = 0.000		SAMN10492703
ALFA	Asian	78	G = 1.00	A = 0.00		SAMN10492704
ALFA	Latin American 2	22	G = 1.00	A = 0.00		SAMN10492700
ALFA	South Asian	8	G = 1.0	A = 0.0		SAMN10492702
ALFA	Latin American 1	6	G = 1.0	A = 0.0		SAMN10492699

### Conservation analysis of *Axin2* (c.1181G > A, p.R394H, rs200899695)

**Fig. 4:**
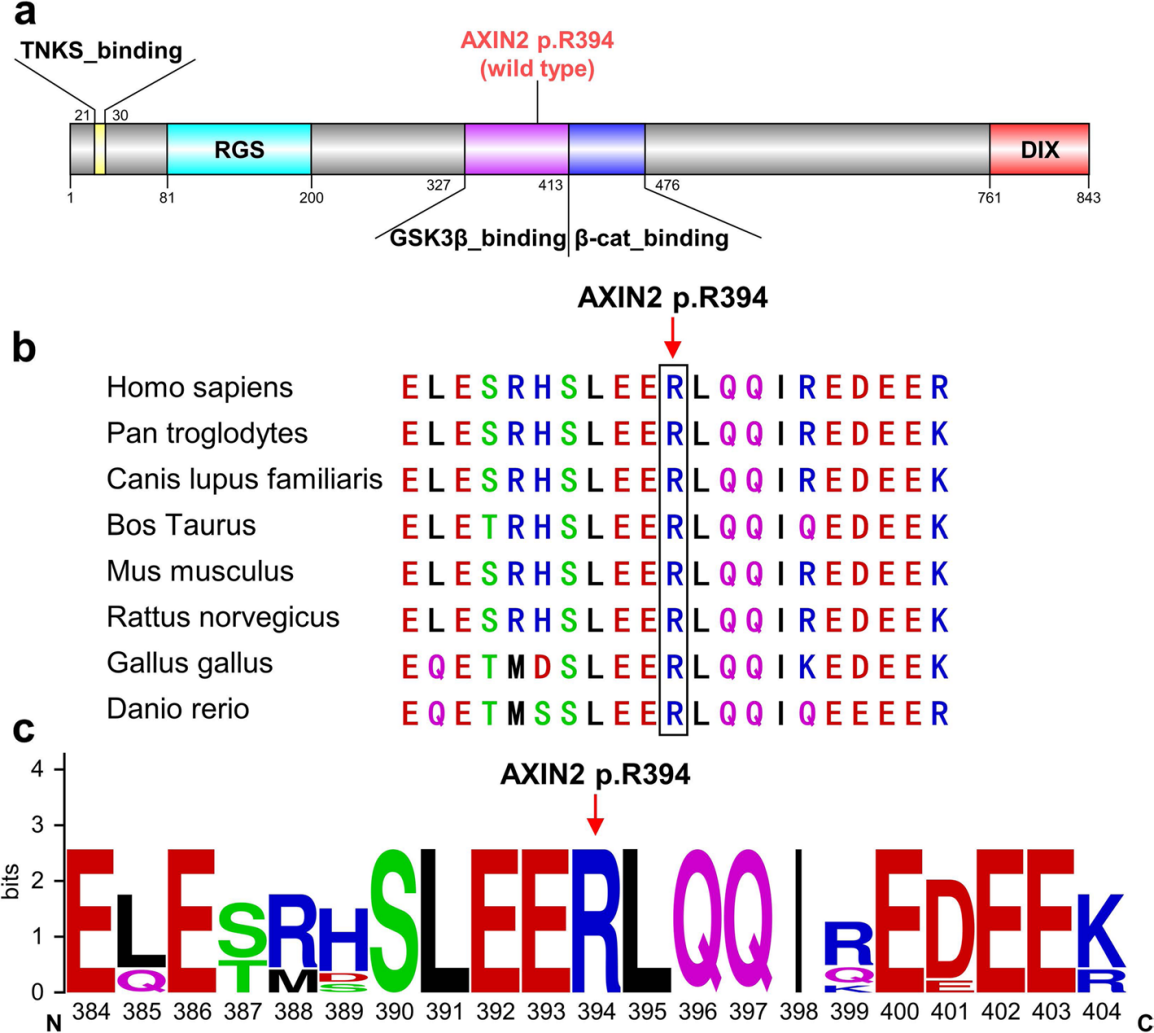
Conservation analysis of *Axin2* (c.1181G > A, p.R394H, rs200899695). **a** Schematic diagram depicts structure of AXIN2 protein. The mutation (R to H at 394^th^ amino acid) resides in AXIN2 GSK3β binding domain. (TNKS_binding: Tankyrase binding N-terminal segment of AXIN; RGS: Regulator of G protein signaling; DIX: Disheveled and AXIN interacting) (**b**) Evolutionary conservation analysis revealed that the Arg394 site is conserved from zebrafish to humans. **c** WebLogo analysis showed that the Arg394 site was relatively conserved

G to A transition of *Axin2* (c.1181G > A, p.R394H, rs200899695) resulted in the replacement of Arg by His at 394^th^ *AXIN2* protein residue (Fig. 4a). Arg394 residue, located at the GSK3β binding domain (amino acid 327 to 413 according to the UniProt Consortium) of Axin2 protein (Fig. 4a) [33], is conserved across species (Fig. 4b,c and Table 2).

**Table 2:** Conservation prediction of *AXIN2* (c.1181G > A: p.R394H, rs200899695)

Conservation prediction	Value	Range ^a^
priPhCons	0.99	0—0.999 (default: 0.0)
mamPhCons	0.97	0—1 (default: 0.0)
verPhCons	1	0—1 (default: 0.0)
priPhyloP	0.42	-10.761—0.595 (default: -0.029)
mamPhyloP	2.75	-20—4.494 (default: -0.005)
verPhyloP	4.82	-20—11.295 (default: 0.042)
GerpN	16.5	0—19.8 (default: 3.0)
GerpS	12.1	-39.5—19.8 (default: -0.2)

^a^ A higher score means the region is more conserved

### Functional annotation and structural analysis and of *Axin2* (c.1181G > A: p.R394H, rs200899695)

**Fig. 5:**
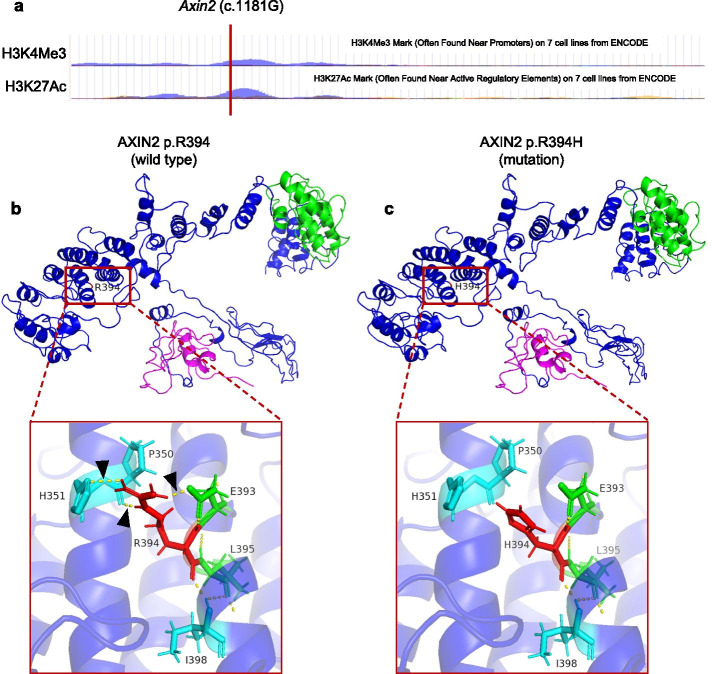
Functional annotation and structural analysis and of *AXIN2* (c.1181G > A: p.R394H, rs200899695). **a** UCSC database predicted that wild type *AXIN2* (c.1181G) loci locates in the region modified by H3K4Me3 or H3K27Ac in seven cell lines by ChIP-Seq assay. **b** Location of Arg394 residues within the GSK3β binding domain of AXIN2. **c** Location of the His 394 residues within the GSK3β binding domain of AXIN2. Arrowheads indicate the hydrogen bond in the domain

*Axin2* (c.1181G > A: p.R394H, rs200899695) mutation is predicted to be potentially deleterious by in silico analysis (Table 3) and the wild type *Axin2* (c.1181G) loci is located within a region modified by H3K4Me3 and H3K27Ac (Fig. 5 a). In addition, p. R394H substitution is predicted to affect spatial structure of AXIN2 GSK3β binding domain (Fig. 5 b, c).

**Table 3:** Functional annotation of *AXIN2* (c.1181G > A: p.R394H, rs200899695)

Block/Annotation Name	Data
**ClinVar**	
Allele Origin	germline
**Variant Category**	
Disruptive Missense ^a^	Yes
GeneHancer ^b^	Yes
SuperEnhancer ^c^	Yes
**Protein Function**	
Polyphen2_HDIV	Probably damaging
Polyphen2_HVAR	possibly damaging
MutationTaster	Disease causing
LRT	Deleterious
SIFT	Deleterious
MutationAssessor	predicted functional (medium)
FATHMM	Deleterious
PROVEAN	Deleterious
MetaSVM	Deleterious
MetaLR	Deleterious
M-CAP	Deleterious
CADD_phred	Deleterious
Fathmm-MKL_coding	Deleterious

^a^ Defined as “disruptive” by the ensemble MetaSVM annotation

^b^ Predicted human enhancer sites from the GeneHancer database

^c^ Predicted super-enhancer sites and targets in a range of human cell types

### Phenotype analysis of *Axin2* knockout mice

**Fig. 6:**
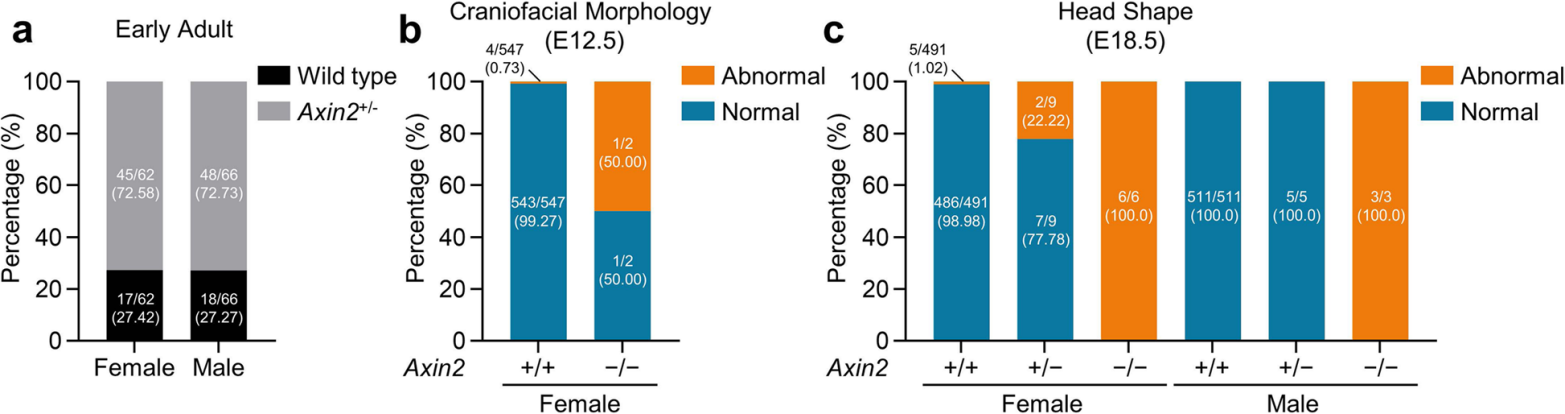
Phenotype analysis of *Axin2* homozygous (*Axin2*^*−/−*^) and heterozygous (*Axin2*^*+/−*^) knockout mice. Data of *Axin2*^*−/−*^ and *Axin2*^*+/−*^ mice were obtained from IMPC. **a ***Axin2*^*−/−*^ mice could not be alive due to preweaning lethality. Number and percentage of *Axin2*^*+/−*^ and matched *Axin2*^*+/+*^ mice at early adult stage. **b** Number and percentage of female mice (*Axin2*^*+/+*^, *Axin2*^*−/−*^) with abnormal craniofacial morphology at E12.5. **c** Number and percentage of mice (*Axin2*^*+/+*^, *Axin2*^*+/−*^, *Axin2*^*−/−*^) with abnormal head shape at E18.5

Data from The International Mouse Phenotyping Consortium (IMPC) documented that homozygous *Axin2* knockout (*Axin2*^*−/−*^) mice developed preweaning lethality, while heterozygous *Axin2* knockout (*Axin2*^*+/−*^) was alive (Fig. 6 a). The percentage of abnormal craniofacial morphology at embryonic day 12.5 (E12.5) for female *Axin2*^*+/+*^ and *Axin2*^*−/−*^ mice was 0.73% (4/547) and 50% (1/2), respectively (Fig. 6 b). In addition, all female (6/6) and male (3/3) *Axin2*^*−/−*^ mice had abnormal head shape, whereas 22.22% (2/9) female *Axin2*^*+/−*^ mice were with abnormal head shape at E18.5 (Fig. 6 c). However, none of male (0/5) *Axin2*^*+/−*^ mice presented with abnormal head shape (Fig. 6 c). Taken together, these results indicate incomplete penetrance of *Axin2* haploinsufficiency in female mice.

## Discussion

Craniosynostosis, a highly heterogeneous disease, is caused by genetic mutations, adverse environmental exposures and their interactions. Identifying the pathoetiology of craniosynostosis gives light to uncover susceptibility individuals, discern environmental risk factors and establish effective strategies for prevention and early diagnosis. In our study, we demonstrated that a heterozygous *Axin2* (c.1181G > A: p.R394H, rs200899695) mutation was presented in the monochorionic twins and their father, but not in the mother. However, only the female proband, who was received additional environmental insults (persistent breech presentation and intrauterine growth restriction), developed sagittal craniosynostosis. We assume that this *Axin2* mutation predisposes to sagittal craniosynostosis but extra environmental insults are needed to initiate the disease.

Prenatal risk factors, including intrauterine constraint, twin gestation, premature delivery, maternal thyroid disorders, gestational diabetes, malnutrition, virus infectious, increase the susceptibility of craniosynostosis in genetically predisposed infants [7, 11–14]. Research of monochorionic (MC) twins provide exceptional opportunity to decipher the interplay between genetic and environment risks on the occurrence of premature suture fusion [34]. In our study, monochorionic twins suffered the majority of risk factors prenatally, however, only the infant with breech presentation and intrauterine growth restriction presented sagittal craniosynostosis. Thus, intrauterine growth restriction and breech position deserves particularly attention in causing sagittal craniosynostosis.

It has been well accepted that AXIN2 is essential for normal calvarial morphogenesis by directly targeting β-catenin, orchestrating the crosstalk of Wnt, BMP, FGF signaling pathways and maintaining suture cell stemness [35–37]. Deletion or mutation of *Axin2* attribute to craniosynostosis in humans and mice [6, 36]. Moreover, phenotype data available in The International Mouse Phenotyping Consortium (IMPC, https://www.mousephenotype.org/) show that all female and male homozygous *Axin2* knockout (*Axin2*^*−/−*^) mice present abnormal head shape malformation. For heterozygous *Axin2* deletion (*Axin2*^*+/−*^) mice, a total of 2/9 females develop abnormal head shape at E18.5; however, the male *Axin2*^*+/−*^ mice are not. These results indicate haploinsufficiency of *Axin2* in female mice with incomplete penetrance.

In this study, an *Axin2* heterozygous missense mutation (c.1181G > A: p.R394H, rs200899695) was identified in peripheral blood samples of subjects I-1, II-1 and II-2 (Fig. 1a), suggesting that the proband inherits the mutation from her father. The wild type *Axin2* (c.1181G) loci is conserved across species and in the region may be modified by H3K4Me3 or H3K27Ac in seven cell lines by ChIP-Seq assay. H3K4Me3 modification is known to mark genes that are essential for the identity and function. H3K4Me3 breadth contains information that ensures transcriptional precision at the identified genes [38]. H3K27Ac, a robust mark of active enhancers and promoters, has been demonstrated to be strongly correlated with gene expression and transcription-factor binding [39]. However, much more functional annotation and experiments are needed to clarify whether *Axin2* (c.1181G > A: p.R394H, rs200899695) could affect the H3K4Me3 or H3K27Ac modification. In addition, *Axin2* missense mutation (c.1181G > A: p.R394H, rs200899695) is likely to be deleterious by in silico predication; however, only the proband received additional risk factors (persistent breech presentation and intrauterine growth restriction) developed sagittal craniosynostosis. We assumed that phenotypic segregation in our case was probably due to *Axin2* (c.1181G > A) mutation possesses incomplete penetrance, thus making it insufficiency to trigger the disease alone. Our findings corroborate another well-established gene-environment interaction model of NCS, which substantiates the same environmental insults ultimately determining phenotype [15]. However, our gene-environment interaction fashion was observed in the context of *Axin2* (c.1181G > A, p.R394H, rs200899695) mutation and female individual, further clinical observations, animal and mechanistic studies are needed to validate the hypothesis.

However, there are some limitations of our study. Experiments on whether *Axin2* (c.1181G > A, p.R394H, rs200899695) mutation could affect the H3K4Me3 or H3K27Ac modification of AXIN2 are needed. We only offered a plausible explanation of *Axin2* (c.1181G > A, p.R394H, rs200899695) mutation and perinatal risk factors contribute to sagittal craniosynostosis in a Chinese female monochorionic diamniotic twin family. Given the small sample size and inherent ascertainment bias, the identification of further families in the setting of this mutation would help to clarify the clinical implications.

## Conclusion

Based on the results of monochorionic twins, we demonstrated *Axin2* (c.1181G > A, p.R394H, rs200899695) mutation led to haploinsufficiency with incomplete penetrance in female, and additional prenatal risk factors (intrauterine growth restriction and breech position) were indispensable to trigger the occurrence of sagittal craniosynostosis in this Chinese female monochorionic diamniotic twin family. These findings provide new evidence for the gene-environment interplay in understanding etiologies of NCS.

## Supplementary Information

**Additional file 1**

## Data Availability

All the data generated in the present research is contained in this manuscript.

## Abbreviations

*Axin2*: Axis inhibitor 2
BMP: Bone morphogenic protein
CS: Craniosynostosis
ExAC: Exome Aggregation Consortium
FET: Frozen embryo transfer
FGF: Fibroblast growth factor
*FGFR2*: Fibroblast growth factor receptor 2
IMPC: The International Mouse Phenotyping Consortium
MAF: Minor allele frequency
MC twins: Monochorionic (MC) twins
MCDA: Monochorionic diamniotic
NCS: Non-syndromic craniosynostosis
TSH: Thyroid-stimulating hormone
*TWIST1*: Twist, drosophila, homolog of 1
WES: Whole exome sequencing
Wnt: Wingless-type integration sites
