# Supplementary material for: An Axin2 mutation and perinatal risk factors contribute to sagittal craniosynostosis: evidence from a Chinese female monochorionic diamniotic twin family
Source: Hereditas. 2021 Jun 16;158:20. doi: 10.1186/s41065-021-00182-0 (PMC8210395; doi:10.1186/s41065-021-00182-0)
Supplement: Supplementary file 1 — Additional file 1 [file 41065_2021_182_MOESM1_ESM.docx]

Supplementary Tables

**Table S1.** Toxoplasmosis, rubella, cytomegalovirus and herpes simplex (TORCH) test result of proband’s mother at 13 weeks of gestation.

| Pathogen | IgM | IgG |
| --- | --- | --- |
| Toxoplasmosis | Negative | Negative |
| Rubella | Negative | Negative |
| Cytomegalovirus | Negative | **Positive** |
| Herpes simplex | Negative | **Positive** |

**Table S2.** Dietary nutrient intake at 14 weeks of gestation.

| Nutrients | Intake | Reference^a^ | | | |
| --- | --- | --- | --- | --- | --- |
|  |  | EER^b^ | RNI^c^ | AMDR^d^ | AI^e^ |
| Macronutrient |  |  |  |  |  |
| Energy (kcal/d) | 1062↓ | 2400 |  |  |  |
| Protein (g/d) | 31.2↓ |  | 70 |  |  |
| Fat (%E/d) | 13.2 |  |  | 20-30 |  |
| Carbohydrate (%E/d) | 75.6↑ |  |  | 50-65 |  |
| Vitamin |  |  |  |  |  |
| VA (μg REA/d) | 701.3↓ |  | 770 |  |  |
| VE (mg α-TE/d) | 24.9 |  |  |  | 14 |
| VB1 (mg/d) | 0.63↓ |  | 1.4 |  |  |
| VB2 (mg/d) | 0.66↓ |  | 1.4 |  |  |
| VC (mg/d) | 288.4 |  | 115 |  |  |
| Folic acid (μg DFE/d) | 318.2↓ |  | 600 |  |  |
| Nicotinic acid (mg NE/d) | 9.4↓ |  | 12 |  |  |
| Mineral |  |  |  |  |  |
| Selenium, Se (μg/d) | 25.5↓ |  | 65 |  |  |
| Calcium, Ca (mg/d) | 454.9↓ |  | 1000 |  |  |
| Iron, Fe (mg/d) | 15.9↓ |  | 24 |  |  |
| Magnesium, Mg (mg/d) | 289.5↓ |  | 370 |  |  |
| Phosphorus, P (mg/d) | 651↓ |  | 720 |  |  |
| Potassium, K (mg/d) | 2556.4 |  |  |  | 2000 |
| Sodium, Na (mg/d) | 2712.8 |  |  |  | 1500 |
| Zinc, Zn (mg/d) | 6.9↓ |  | 9.5 |  |  |
| Copper, Cu (mg/d) | 2.3 |  | 0.9 |  |  |
| Manganese, Mn (mg/d) | 4.7 |  |  |  | 4.9 |
| Iodine, I (μg/d) | 435.8 |  | 230 |  |  |

^a^Reference data were from Chinese Dietary Reference Intakes (DRIs), 2013.

^b^EER: estimated energy requirement.

^c^RNI: recommended nutrient intake.

^d^AMDR: acceptable macronutrient distribution ranges.

^e^AI: adequate intakes.

**Table S3.** Thyroid function tests and urinary iodine level at 17 weeks of gestation.

| Item | Value | Reference |
| --- | --- | --- |
| Serum free Triiodothyronine, fT3 (pg/mL) | 2.75 | 2.50-3.90 |
| Free Thyroxine, fT4 (ng/dL) | 0.82 | 0.61-1.12 |
| Thyroid-stimulating hormone, TSH (μIU/mL) | 0.12↓ | 0.34-5.10 |
| Urinary iodine (μg/L) | 383.86↑ | 150-250 |

**Table S4.** Glucose tolerance test at 27 weeks of gestation.

| Item | Value | Reference |
| --- | --- | --- |
| Fasting blood glucose level (mmol/L) | 7.38↑ | 3.7-6.1 |
| Postprandial one-hour blood glucose level (mmol/L) | 15.30↑ | <11.1 |
| Postprandial two-hour blood glucose level (mmol/L) | 11.03↑ | <7.8 |


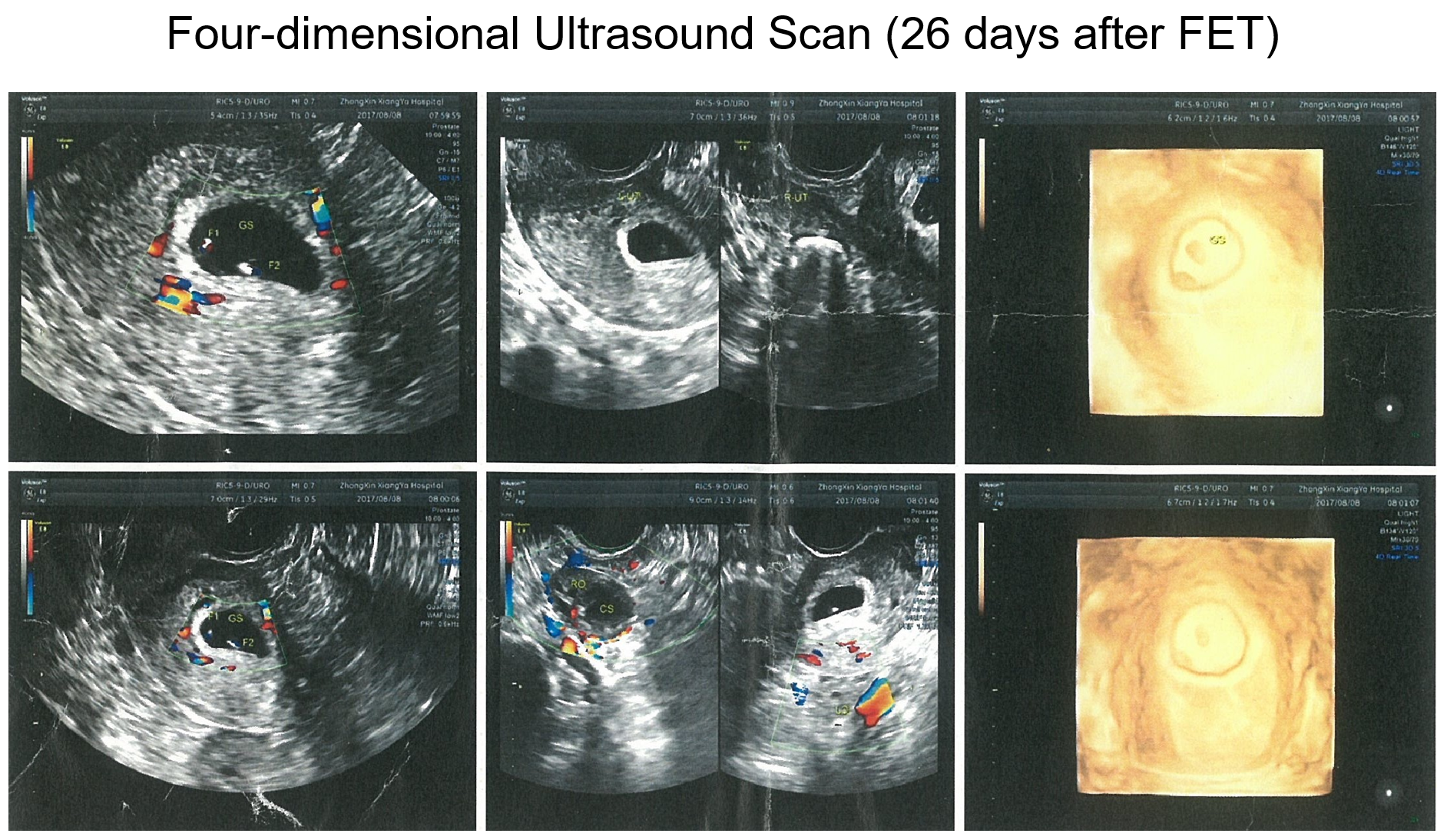


**Fig. S1.** Pictures of four-dimensional ultrasound scan of the infants at 26 days after FET. The monochorionic diamniotic (MCDA) twins were diagnosed.


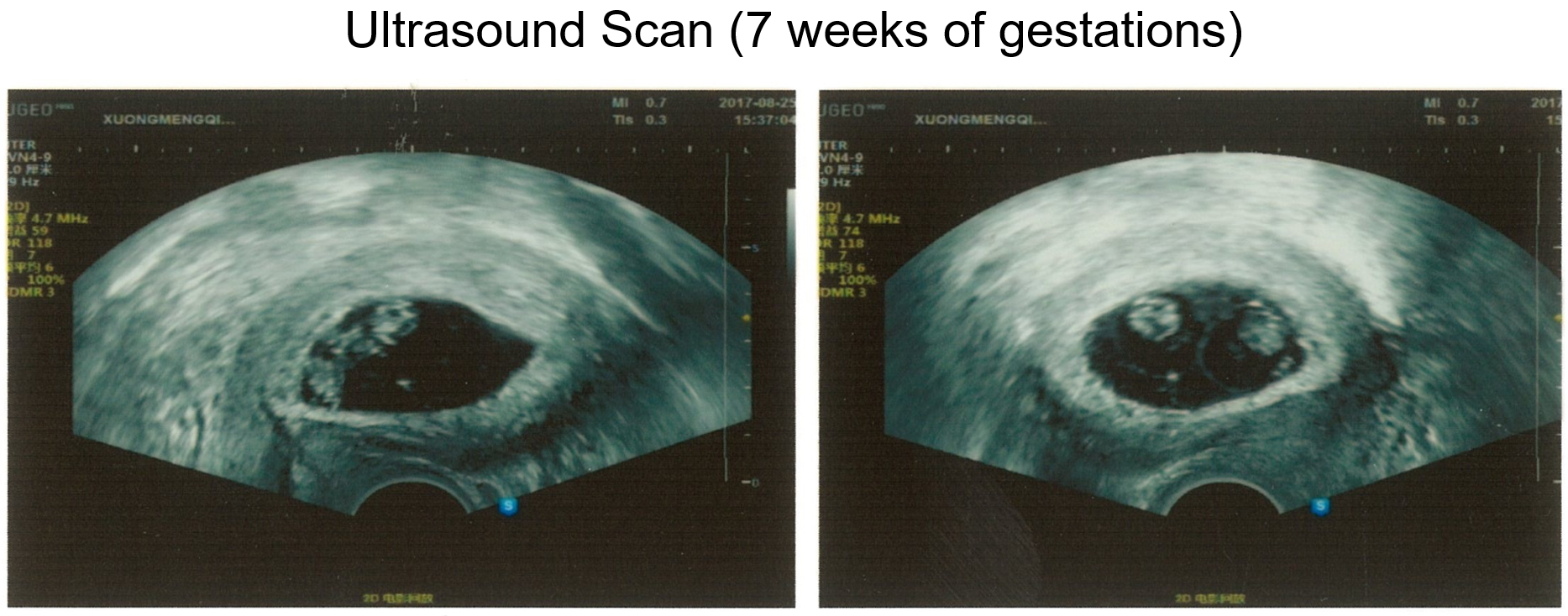


**Fig. S2.** Pictures of ultrasound scan of the infants at 7 weeks of gestations. Two yolk sacs and two embryos with heart beats were detected.


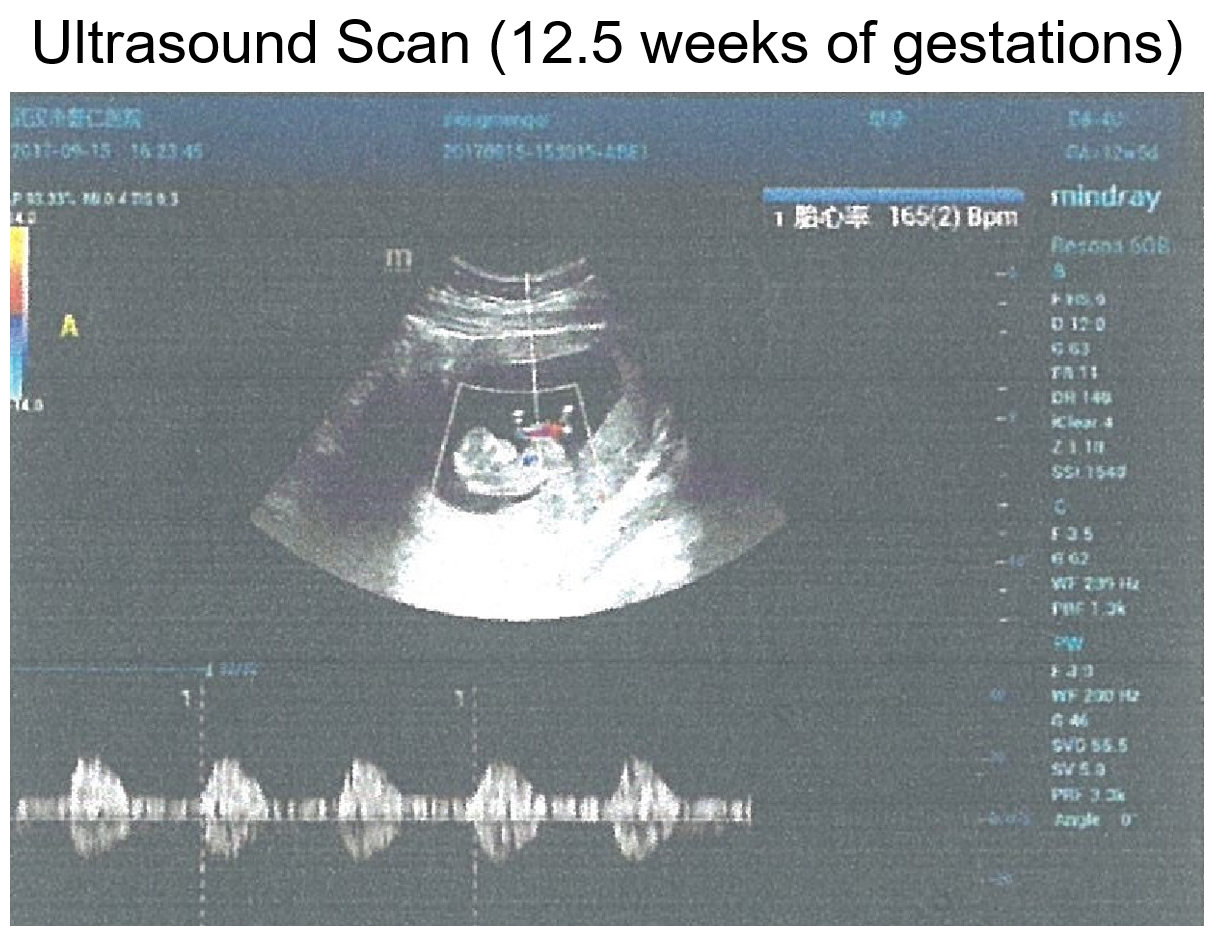


**Fig. S3.** Pictures of ultrasound scan of the infants at 12.5 weeks of gestations.
